# Synopsis of congenital cardiac disease among children attending University of Nigeria Teaching Hospital Ituku Ozalla, Enugu

**DOI:** 10.1186/1756-0500-6-475

**Published:** 2013-11-19

**Authors:** Josephat M Chinawa, John C Eze, Ikechukwu Obi, Ijeoma Arodiwe, Fortune Ujunwa, Adiele K Daberechi, Herbert A Obu

**Affiliations:** 1 Department of Paediatrics, University of Nigeria/Teaching Hospital, Enugu, Nigeria; 2 University of Nigeria/Teaching Hospital, Enugu, Nigeria; 3 University of Nigeria, Enugu Campus, and University of Nigeria Teaching Hospital, Ituku Ozalla, Enugu, Nigeria

**Keywords:** Congenital cardiac disease, Prevalence, Pattern, Children, Enugu

## Abstract

**Background:**

The objective of this study was to determine the pattern of congenital cardiac disease among children attending UNTH, Enugu, Nigeria. The nature of these abnormalities and the outcome were also considered. The exact etiology is unknown but genetic and environmental factors tend to be implicated. The difference in the pattern obtained worldwide and few studies in Nigeria could be due to genetic, environmental, socioeconomic, or ethnic origin.

**Methods:**

A retrospective analysis of discharged cases in which a review of the cases of all children attending children outpatient clinics including cardiology clinic of the University of Nigeria Teaching Hospital (UNTH), Enugu over a five year period (January 2007-June 2012) was undertaken. All the children presenting with cardiac anomalies were included in the study and the cases were investigated using ECG, X-ray and echocardiography studies.

**Results:**

A total of 31,795 children attended the children outpatient clinics of the hospital over the study period. Of these, seventy one (71) had cardiac diseases. The overall prevalence of cardiac disease is 0.22%. The commonest symptoms were breathlessness, failure to thrive and cyanosis. Almost all types of congenital detects were represented, the commonest being isolated ventricular septal detect (VSD), followed by tetralogy of Fallot. One of these cardiac anomalies presented with Downs’s syndrome and another with VACTERAL association.

**Conclusions:**

The results of this study show that 0.22% per cent of children who attended UNTH in Enugu State had congenital cardiac abnormalities and the commonest forms seen were those with VSD.

## Background

Cardiac disease is the leading cause of morbidity and mortality in the United States for the past 80 years [1]. This may not be the same in developing countries, where malaria and malnutrition are major culprits [2]. Notwithstanding, mortality and morbidity from cardiac disease among children in developing countries are gaining recognition. Heart disease also results in substantial health-care expenditures and in USA for example, coronary heart disease was projected to cost an estimated US $151.6 billion in 2007 [3].

Of heart diseases in children, congenital heart defect is commoner, affecting 8 in every 1000 live births, and accounting for >20% of perinatal deaths [4]. There is a high incidence of chromosomal abnormalities that are associated with CHD [5,6].

Over the last 15 years, 1.35 million newborns with CHD were born every year. Prevalences from this number vary worldwide [7]. For instance, Asia reported the highest CHD birth prevalence, with 9.3 per 1,000 live births with relatively more pulmonary outflow obstructions and fewer left ventricular outflow tract obstructions [7]. Reported total CHD birth prevalence in Europe was significantly higher than in North America (8.2 per 1,000 live births vs. 6.9 per 1,000 live births). Access to health care is still limited in many parts of the world, as are diagnostic facilities, probably accounting for differences in reported birth prevalence between high- and low-income countries. In addition, the difference in the pattern obtained worldwide and few studies in Nigeria could be due to genetic, environmental, socioeconomic, or ethnic origin, and there need to be further investigation to tailor the management of this global health problem.

The factors implicated in congenital cardiac disease in children are usually both genetic and environmental, where a combination of genes from both parents with unknown environmental factors, produce the trait or condition.

Prevalence studies of congenital cardiac disease are necessary to establish baseline rates, to establish changes over time and to identify clues to etiology. They are also important for health services planning and evaluating antenatal screening in populations with high risk. The study is also important as it may help to raise the awareness of surgical intervention in childhood and to emphasize the loss of babies with cardiac abnormalities.

We are not aware of any study among pediatric age from Enugu or South-East Nigeria in general. In addition, the UNTH moved to its permanent site at Ituku-Ozalla five years ago and since then no work has been done on the prevalence and pattern of presentation of congenital abnormalities in newborns in the area. This study was thus designed to bridge this gap with a view to determining the prevalence of cardiac abnormalities among children attending UNTH, Ituku-Ozalla, Enugu. It is hoped that this will add to the body of knowledge available on these disorders and may stimulate further research in the area on the subject.

## Methods

The study was conducted at the children outpatient clinic of the UNTH, Ituku/Ozalla, Enugu. The hospital was located at her temporary site within the city (Enugu) centre. In January 2007, the hospital was re-located to its permanent site at Ituku/Ozalla, 22 kilometers away from Enugu metropolis. The hospital provides care for children and also receives referrals from different parts of Enugu, the rest of Enugu State and surrounding states.

A retrospective analysis of all discharged cases were carried, in which a review of the records of all children attending UNTH over a five year period (January 2007 to April 2012) was undertaken. The folders (case files) of these children were retrieved from the hospital records department. Data collection was done with structured Questionnaire. This questionnaire design contain information on age, sex, diagnosis by 2D Echocardiogram, address, social class among others. Also obtained from the folders was clinical history, which included antenatal history, history of exposure to teratogens and family history of consanguinity.

The diagnosis of congenital cardiac abnormality was based on clinical evaluation and 2 D-echocardiography (as documented by doctors in the patients’ folders).

In this study we classify isolated ventricular septal defects, isolated atrial septal defects and isolated pulmonary stenosis as abnormalities that do not coexist with any other cardiac anomaly. We also classify complex cardiac anomaly as a set of associated malformations involving parts that are necessary for maintenance of the patient’s life, each of them being classified as follows: total anomalous pulmonary venous drainage, hypoplastic left heart syndrome, single ventricle, mitral atresia, pulmonary atresia with intact ventricular septum, tricuspid atresia, double right ventricular outflow tract, double left ventricular outflow tract, tetralogy of Fallot, truncus arteriosus, and transposition of the great vessels) [8]. These defects may coexist with other cardiac lesions.

The prevalence rate of congenital cardiac disease was estimated as arithmetic percentage of the total number of children attending children outpatient within the period of the study.

Subjects whose ages were between 6months and 18 years were included in this study, while those subjects whose diagnoses were proven by means of 2-D Echocardiography were also included. Subjects with incomplete data were excluded from the study.

The families were assigned socioeconomic classes using the recommended method (modified) by Oyedeji [9]. The parents’ occupation and highest education attained were scored from 1 (highest) to 5 (lowest). The mean score for both parents gives social class falling within the 1–5 range. Those with the mean score of <2 were further reclassified into upper class while those with the mean score of >2 were reclassified into lower social class. For the occupation score, those in upper social class included parents, such as senior public officers, large-scale traders, large-scale farmers and professionals. Lower class included artisans, primary school teachers, peasant farmers, labourers and the unemployed. For the education score, those with PhD, master degree, bachelor degree and higher national diploma (HND) were categorized as upper class. Those with ordinary national diploma (OND), national certificate of education (NCE), technical education, grade II teachers’ certificate, junior and senior secondary school certificate, primary school certificate and those with no formal education were classified as lower social class [9].

Use was made of SPSS 13 in other data analysis. Rates and proportions were calculated with 95% confidence intervals. The proportions were compared using students T-test. Level of significance was set at P< 0.05.

Ethical approval for this study was sought from the Ethics and Research Committee of UNTH.

The aims and objectives of this study were to determine the prevalence of cardiac abnormalities among children attending the children outpatient clinic of the UNTH, Ituku-Ozalla, Enugu State; to describe the different forms of abnormalities seen among these children; to determine the various clinical profile, and outcome of cardiac abnormality among children in UNTH.

## Results

### Demography

A total of 31,795 children attended the children outpatient clinic of the hospital over the study period. Of these; seventy one (71) had congenital cardiac disease, giving a prevalence of 0.22 per cent.

Out of the 71 children with cardiac disease, 35 (49.30%) were males while 36 (50.70%) were females, giving a female: male ratio of 1:1. The children were aged 6 months to 12 years. The mean age of the children was 7.82 ± 4.12 years. The most common age group in this study was the under five which represented 57.7% of children with congenital cardiac disease. Majority of the parents of children with congenital cardiac anomaly were of very low socioeconomic class as shown in Table 1.

**Table 1:** Socio-demographic characteristics of respondents

	**Frequency N = 71**	**Percent**
Age range		
1 – 6	41	57.7
7 – 12	22	31.0
13 – 18	8	11.3
Sex		
Female	36	50.7
Male	35	49.3
Social class		
SEC 1	1	1.4
SEC2	12	16.9
SEC3	16	22.5
SEC4	21	29.6
Unknown	21	29.6

Among children who presented with congenital cardiac disease, isolated ventricular septal defect (VSD) occurred in 14 (21.5%) of them. Isolated VSD is both the commonest cardiac disease overall and acyanotic congenital heart disease in particular. Tetralogy of Fallot (TOF) accounted for 6 (9.2%) children and is the commonest cyanotic congenital heart disease. This is shown in Table 2.

**Table 2:** Echocardiography findings

	**Frequency N = 71**	**Percent**
	21	29.6
Isolated VSD	14	19.8
TOF	9	12.7
RHD	4	5.7
AVSD	3	4.2
TA	3	4.2
Isolated ASD	2	2.8
CS	2	2.8
DORV	2	2.8
ECD	2	2.8
PDA	2	2.8
HRV	1	1.4
CMS	1	1.4
Dextrocardia	1	1.4
SV	1	1.4
Isolated PS	1	1.4
TAPVD	1	1.4
DC	1	1.4

Table 3 shows that majority of mothers of 40 (56.3%) of children with cardiac disease did not expose themselves to any drug or irradiation while few 3 (4.2%) took herbal concoction. A good number 36 (50.7%) took their routine ante natal drugs. Three (4.6%) of the children with congenital cardiac anomaly had VACTERAL association and 4 (6.2%) had syndromes; especially Down’s syndrome and Turners syndrome.

**Table 3:** Maternal drug intake and associated syndromes

	**Frequency N = 61**	**Percent**
Herbal Drug use **(Dogo yaro)**	3	4.2
**Routine drugs only (Folic acid 1 tab daily and fesolate 1 daily with multivitamins 1 daily) especially at the first trimester**	36	50.7
No alcohol use	40	56.3
No smoking	40	56.3
No exposure to Irradiation	38	53.5
***Presence of congenital malformation***	10	14.1
***Type of association***		
VACTERAL associations	3	4.2
***Associated syndromes***		
No	37	52.1
Yes	4	5.6
***Specific syndrome***		
Down Syndrome	2	2.8
Turner Syndrome	1	1.4

As depicted in Table 4, only 4 (6.2%) children had a form of surgery, while 40 (61.5%) defaulted for various reasons but 21 (32.3%) were on follow up.

**Table 4:** Outcome

	**Frequency**	**Percent**
Cardiac catheterization	1	1.4
Cardiac surgery for PDA	2	2.8
VSD repair + PDA ligation	1	1.4
	Frequency N = 71	Percent
Currently followed up	21	29.6
Defaulted	40	56.3
No records	10	14.1

## Discussion

Congenital heart anomaly is defined as structural abnormality of heart or adjacent great blood vessels present either at the time of birth or detected later in life while the acquired is triggered by insults on the heart after birth. The exact etiology is unknown but genetic and environmental factors tend to be implicated. Of cardiac diseases in childhood, congenital heart disease (CHD) occurs more frequently than acquired heart disease [7]. In developing countries, CHD is one of the non communicable diseases that cause mortality and morbidity in children.

The prevalence of congenital heart defect is 0.5-0.8% live birth and increases to 2-6% if first degree relative is affected. The prevalence of heart disease in general among children is 8 per 1000 live births. The prevalence of 0.22% obtained in our study is similar to that reported by Nelson et al. [8] who noted a prevalence rate of 2 and 10 per 1000 live births. The observed similarities in prevalence with studies that looked at populations not similar to ours could be due to the fact that both studies were done in a referral institution where major cardiac defects are seen.

Ejim and colleagues [10] evaluated the prevalence of ventricular septal defect (VSD) among patients referred for echocardiographic examination at the echocardiography laboratory of the University of Nigeria Teaching Hospital, Enugu, Nigeria over a 10-year period. They noted that of 2486 echocardiogram scans done; 593 subjects had congenital anomalies, of which 165 (prevalence of 0.28) had VSDs. This is also similar to our findings. From this study, there exists a male predominance when congenital heart disease is considered.

It is noted with interest, that most of the children with congenital cardiac anomaly were from very low socioeconomic background. Hemingway [11] and colleagues in his study also noted a strong association between low socioeconomic class and cardiac disease. The reason for this could be adduced from the fact that mothers from high socioeconomic class will seek corrective surgery abroad and as such may not report at local hospitals as against the low socioeconomic ones who cannot afford money for cardiac surgery.

The mean maternal age (in years) of those with cardiac anomaly is 32.3 ± 6. Grag [12] and colleagues also noted a high occurrence of congenital abnormality and other cardiac defects among women who are between 33 and 39 years of age. Other workers [12,13] in their studies suggested that high pregnancy rates among mothers in this age range could account for the congenital abnormality.

Consanguineous marriage is an important factor that contributes to congenital cardiac disease among children. This is influenced by the degree of relatedness between the spouses such as first cousins, double first cousins and second cousins [14]. A consistent positive correlation has been reported between consanguinity and VSD, atrial septal defect (ASD), pulmonary atresia, tetralogy of Fallot and other CHDs in different populations [15]. We did not obtain any correlation between consanguinity and congenital heart disease in this study. Consanguinity, however, is not a common practice among the Igbos who are the indigenous and predominant inhabitants of Enugu and environs.

Isolated ventricular septal defect, with a frequency of 19.8%, was the most common defect in our study. This is lower than that reported in a recent study 41.59%, but higher than the frequency reported in another study [16,17]. Factors such as method of diagnosis, non recognition of minimum or small septal defects by the physician responsible for primary care and poor hospital visits due to poverty and ignorance, may have contributed to the difference between our figures and those in the literature. With respect to cyanotic congenital heart defects, tetralogy of Fallot (8.5%), tricuspid atresia (4.2%) and total anomalous pulmonary venous drainage (1.4%) were the most frequent anomalies. According to the literature, the most prevalent anomaly is transposition of the great vessels, with an incidence ranging from 3.5 to 10.9% [18]. We recorded no case of TGA in our study, this is because death could have occurred before getting to a health facility and thus could not be accounted for. From this study, mothers of children with cardiac anomaly are relatively young, with very few taking local concoction and alcohol. The commonest local concoction taken by pregnant women in Nigeria contain aqueous *bitter leaf, ginger, agbo, dogo yaro* and *cannabis*[19]. Some of these herbal concoctions could cause deleterious effects in pregnancy. For instance Fischer-Rasmussen *et al.*[20] noted that ginger has been associated with mutagenesis in a culture of Escherichia coli *(E.coli)*.

We could not conclude if there is any relationship between maternal intake of local concoction and congenital heart anomaly. Nevertheless, only three mothers took *dogo yaro* (a local concoction known to contain quinine) as a tonic to treat malaria during first trimester. Al-alni et al. [21] in his Saudi Arabia study noted no relationship between cardiac anomaly with maternal age and gender. This is in agreement with our study where sex ratios were equal.

It is important to note that VACTERAL associations and talipe deformities were seen in some children with congenital cardiac anomaly in our study. VACTERAL associations and talipe deformity have been reported in some children with cardiac anomaly by some workers [22]. Martinez-Frias et al. [23] in his work noted tetralogy of Fallot as the most common congenital heart defects seen in the VACTERAL association. It is noted in this present study that VSD and AVSD are associated with Down’s syndrome. This was corroborated by Paladini [24] and his workers who noted an incidence of 56% and 44% respectively among fetuses with AVSD and VSD, using amniocentesis and karyotyping. Dawson et al. [25] in a study had associated persistent left superior vena cava, coarctation of the aorta and partial anomalous pulmonary venous drainage with Turner’s syndrome. He also suggested jugular lymphatic obstruction in-utero, resulting in dilated lymphatic vessels around the aortic root, which may compress developing outflow structure as a cause of aortic deformation commonly seen in Turner’s syndrome.

It is disheartening to note that only a handful (6.2%) of children with congenital heart disease had surgery and a good number defaulted follow up. Treatment of children with CHD are limited to larger cities and it is quite expensive and beyond the reach of the poor. So many parents could not afford this cost and they will either abandon routine hospital visits or leave their children in the hands of fate.

To improve the survival of children with cardiac disease, there is need to diagnose and treat them at earliest age through provision of affordable diagnostic and surgical as well as other interventional facilities at each of the six geo-political zones of the country.

The main focus of investigation is 2-D echocardiography and ECG. Although trans thoracic echocardiography (TTE) with Doppler has been shown to be of great value in diagnosis of patients with Cor triatriatum; biplanar trans esophageal echocardiogram (TEE) provides a more complete and detailed data of the anatomy of cor triatriatum and other complex cardiac anomalies [26]. In this study, 2-D echocardiography, ECG and chest radiography to were used for the diagnosis of congenital cardiac anomaly.

Prenatal diagnosis may help detect this anomaly early and avert significant morbidity and numerous mortalities that follow this disease. Prenatal diagnosis has not to yet gained ground in the management of congenital cardiac disease in Nigeria.

Prenatal diagnosis allows for extensive parental counseling and coordination of care. The fetal diagnosis of CHD identifies chromosomal abnormalities, which are found in 10% of cases, and assesses associated extra cardiac anomalies, which coincide in 15% of cases [25]. In cases of poor prognosis, early prenatal diagnosis gives the parents the option of coming to a fully informed decision as to whether to continue with the pregnancy in some countries [12].

## Conclusions

Congenital cardiac abnormalities occur in 0.22% of children attending UNTH and the commonest form seen is VSD. The prevalence rate obtained in this study, however, may not reflect the true situation in the general population for reasons adduced in the discussion above but gives a clue to the existence of the problem and could serve as a stimulus for further studies on the subject.

## Competing interests

The authors hereby declare that we have no competing interests.

## Authors’ contribution

All the authors made substantial intellectual contributions to this study. CJM was involved in the conception, design and data collection as well as interpretation of results, preparation of the manuscript, revision of the article at various stages and preparation of the final draft. JCE contributed in conception, design, manuscript preparation and approval of the final document. IEO, IOA, UFA, OHA and AKB made substantial contributions in the design, data collection and interpretation of results as well as the approval of the final document. All authors read and approved the final manuscript.
